# Supplementary material for: Dynamic Changes in Plant Secondary Metabolites Induced by Botrytis cinerea Infection
Source: Metabolites. 2023 May 13;13(5):654. doi: 10.3390/metabo13050654 (PMC10223084; doi:10.3390/metabo13050654)
Supplement: Supplementary file 1 [file metabolites-13-00654-s001.zip › metabolites-2360228-supplementary.pdf]

Table S1 Analysis of Gas chromatography / quadrupole time-of-flight mass on differential non-volatile metabolites of grape 'Victoria' that was either non-inoculated (NI), inoculated with *Botrytis cinerea* B05.10 (B5), or inoculated with *B. cinerea* 242 (B2)

**Compound**		**Retention Time**	**Mass**	**Log_2FC (B5 – NI]**	**Log_2FC (B2 - NI]**
Saccharides	D-(-)-Erythrose	12.7	73.0475	13.1	16.4
	D-(-)-Ribofuranose	13.6	73.0475	14.33	14.4
	D-(-)-Rhamnose	14.9	117.0382	-1	-18.8
	L-(-)-Sorbose	16.6	201.1037	3.4	21.6
	D-(-)-Fructose	16.7	307.1468	15.9	16.7
	D-Glucose	16.8	103.0574	3.5	23
	D-(+)-Talose	16.9	217.1086	3.5	-21.4
	4-Ketoglucose	19.8	73.0475	16.9	-0.2
	Glyceryl-glycoside	20.4	204.1014	0.4	-14.3
	D-(+)-Cellobiose	20.5	204.1013	-0.6	-1.1
	Maltose	21	204.1013	-0.9	-1.7
	2-α-Mannobiose	21.1	204.1013	-1.2	-2.6
	D-Ribose	21.1	205.1065	3.5	17.6
	D-Glucopyranose	21.4	204.1015	11.4	10.9
	D-Psicose	23.9	307.1423	-1	-1.4
	D-Trehalose	24.2	217.1086	14.7	14.6
	β-Gentiobiose	25	361.1725	2.6	2.8
	Palatinose	29.3	361.1726	19.6	-0.2
Amino acid	L-Valine	6.5	72.0388	-2.6	-23
	L-Threonine	9.5	73.0475	-2.3	-3.4
	L-Threitol	12.4	73.0475	1.7	1.2
	L-5-Oxoproline	12.5	156.0755	22.1	14.7
	Phenylalanine	12.7	120.0832	-18.9	-22.5
Organic acid	2,6-Dihydroxybenzoic acid	7.4	267.1121	-3.4	13.4
	Phosphoric acid	7.9	241.0994	-12.7	3.2
	Butanoic acid	10.3	73.0475	19.7	9.5
	Butanedioic acid	10.6	147.0669	-1	-0.61
	Pipecolic acid	12.5	156.0816	-20.4	-20
	γ-aminobutyric acid	12.5	174.1079	23.4	15.6
	N-Acetylaspartylglutamic acid	12.5	304.1394	-14.1	-17.7
	Arabinonic acid	13.8	73.0475	17.7	15.9
	2-Keto-l-gluconic acid	15.5	73.0475	-2	-2.4
	D-(+)-Glucuronic acid	16.1	73.0475	14.6	13.9
	2-Ketoglutaric acid	16.6	319.1606	1.1	1.3
	Galactaric acid	17.8	73.0475	16.2	16.3
	Gluconic acid	19.5	73.0475	17.8	13.6
	1-Aminocyclopentanecarboxylic acid	20.6	204.1012	-7.1	-17.6

Compound		Retention Time	Mass	Log_2FC (B5 – NI]	Log_2FC (B2 - NI]
	cis-Coutaric acid	22.8	219.0944	-1	-1.3
Alcohol	D-(+)-Arabitol	14.5	129.0707	-9.7	-16.7
	D-Mannitol	17.1	204.1013	-16.1	-16.4
	D-Glucitol	21.1	73.0475	0.3	1.3
	Maltitol	27.2	73.0475	-0.8	-1
	Betulin	28.8	216.0913	18.4	17.5
Ester	Dimethyl tartarate	12.9	73.0475	-1.3	-0.7
Phenol	3-Pyridinol	7.2	152.0532	21	13.8
	Arbutin	22.7	73.0475	12.1	12.3
	Resveratrol	23.5	444.2012	2.8	0.9
	Aucubin	23.8	361.1722	-0.5	-11.6
	trans-Piceid	26	354.1563	19.9	12.5
	cis-Piceid	27.7	444.2025	2.6	1.9
Other	Cyclohexasiloxane	9.8	341.0213	-3.2	13.1
	β-Lyxopyranose	14.1	147.0668	-17.1	-17.3
	Copper phthalocyanine	26.7	202.1044	20.9	-0.2

Table S2 Analysis of Gas chromatography / quadrupole time-of-flight mass on differential non-volatile metabolites leaves of grape 'Shine Muscat' that was either non-inoculated (NI), inoculated with *Botrytis cinerea* B05.10 (B5) or inoculated with *B. cinerea* 242 (B2)

**Compound**		**Retention Time**	**Mass**	**Log_2FC (B5 - NI)**	**Log_2FC (B2 - NI)**
Saccharides	4-Ketoglucose	14.6	73.0474	3.2	16
	L-(-)-Sorbose	16.5	349.1506	-0.1	16.4
	D-Allose	19.2	73.0474	-0.1	16.2
	D-(+)-Cellobiose	20.8	204.1013	-3.6	14.5
	Glucose	21.3	204.1017	-0.1	16.7
	D-Glucopyranose	21.4	204.1013	-0.1	17
Amino acid	L-Serine	9	116.0545	16	-0.7
	L-Threonine	9.5	73.0474	13.5	-0.7
Organic acid	γ-aminobutyric acid	12.5	174.1062	-0.1	15.8
	Arabinonic acid	13.8	73.0474	-0.1	16.9
	D-Glucopyranosiduronic acid	21.9	73.0474	-0.1	15.4
	Mandelic acid	25.3	297.1329	-0.1	12.5
Alcohol	3-Methoxythiophenol	10.2	213.0726	-0.1	15.9
	L-Threitol	12.2	73.0474	3.1	15.7
	Glycerol	12.6	73.0475	13.4	-0.7
	Betulin	28.8	216.0922	-0.1	17.7
Ester	D-(+)-Ribono-1,4-lactone	14.3	73.0474	-0.1	12.4
Phenol	Resveratrol	23.5	444.2012	2.762733	2.6
	cis-Piceid	27.7	444.2025	1.4	1.6

Table S3 Differential metabolites compared grapes varieties ‘Shine Muscat’ and ‘Victoria’ that were either inoculated with *Botrytis cinerea* B05.10, inoculated with *B. cinerea* 242, or non-inoculated

Grape and inoculation	Compound	Retention Time	Mass	Regulation	Log_2FC
S-NI & V-NI	Lactic Acid	6.1	147.0670	up	1.1
	L-Serine	8.9	116.0551	down	-23.8
	L-Threonine	9.4	73.0475	down	-23.8
	Oxalic acid	10.4	147.0668	down	-22.5
	Shikimic acid	15.7	204.1013	down	-1.8
	β-D-Glucopyranose	20.5	204.1013	down	-22.4
	Ethyl α-D-glucopyranoside	20.5	204.1013	down	-23.1
	Maltose	21.0	204.1013	down	-23.3
	Hexopyranose	22.4	73.0475	down	-22.7
	Catechine	24.6	368.1678	down	-22.8
S-B5 & V-B5	Lactic Acid	6.1	147.0671	down	-22.4
	L-Serine	9.0	174.1079	down	-22.5
	Ethanolamine	9.1	174.1009	up	23.8
	Phosphonic acid	9.2	299.0739	up	1.2
	Glyceric acid	10.0	73.0474	up	1.3
	Butanedioic acid	10.5	147.0671	down	-22.5
	L-Aspartic acid	11.1	73.0475	down	-22.5
	Pipecolic acid	12.5	84.0439	down	-23.8
	γ-aminobutyric acid	12.5	174.1079	down	-22.9
	Tartaric acid	13.2	73.0474	up	26.1
	Arabinonic acid	14.9	73.0475	up	1.0
	D-(-)-Tagatofuranose	15.3	73.0474	up	1.2
	N-Acetyl glucosamine methoxime	15.5	73.0475	down	-21.9
	Fructofuranoside	15.6	73.0475	down	-22.1
	Shikimic acid	15.7	204.1015	down	-1.8
	D-Fructose	16.5	217.1087	down	-26.3
	D-(-)-Fructose	16.6	307.1459	down	-24.5
	D-Mannitol	17.0	319.161	down	-25.3
	Palatinose	17.3	73.0475	down	-23.2
	β-D-Glucopyranose	20.5	204.1018	down	-22.1
	Ethyl α-D-glucopyranoside	20.6	204.1018	down	-22.4
	D-glucose-6-phosphate	20.7	387.146	up	1.3
	Resveratrol	23.3	169.0692	down	-19.2
	D-Trehalose	24.1	73.0474	up	18.7
	Catechine	24.6	368.1694	down	-1.1
	β-Gentiobiose	25.0	361.1732	down	-22.4

Grape and inoculation	Compound	Retention Time	Mass	Regulation	Log_2FC
	Epigallocatechin	25.1	456.2044	down	-21.9
	Galactinol	25.7	204.1015	up	1.2
S-B2 & V-B2	L-Serine	9.0	174.1056	up	22.8
	1,3-Propanediol	10.4	147.0671	down	-22.5
	Malic acid	12.1	147.0671	down	-20.7
	Pipecolic acid	12.5	84.0436	up	2.3
	Tartaric acid	13.2	73.0474	up	18.3
	L-(+)-Tartaric acid	13.9	294.1348	down	-23.5
	2-Keto-l-gluconic acid	15.4	292.1368	up	1.2
	Fructofuranoside	15.6	217.1085	down	-22.8
	Shikimic acid	15.7	204.1015	down	-1.5
	2-Ketoglutaric acid	16.6	129.071	up	16.7
	d-Galactose	16.7	73.0474	up	1.9
	Palatinose	17.3	73.0476	down	-17.2
	β-D-Glucopyranose	20.5	204.1015	down	-22.2
	cis-Coutaric acid	23.7	219.094	up	18.6
	D-(+)-Turanose	23.9	307.1408	up	23.0
	L-Alanine	24.7	307.1408	up	20.8
	Epigallocatechin	25.1	456.204	down	-22.2
	Isomaltose	25.1	73.0475	down	-17.2
	Epigallocatechin	25.2	456.204	down	-22.9

Grapes treatments included ‘Victoria’ non-inoculated (V-NI), ‘Victoria’ inoculated with *Botrytis cinerea* B05.10 (V-B2), ‘Victoria’ inoculated with *B. cinerea* 242 (V-B2), ‘Shine Muscat’ non-inoculated (S-NI), ‘Shine Muscat’ inoculated with B05.10 (S-B5), and ‘Shine Muscat’ inoculated with 242 (S-B2).

Table S4 Differential volatile metabolites of grape ‘Shine Muscat’ leaves by comparing the group inoculated with *Botrytis cinerea* and the group without inoculation

Days after treatment（inoculated vs non-inoculated groups）	**Compound**	**Time**	**M/Z**	**Log_2FC**	**Regulation**
3 d	β-Pinene	12.0	93.0699	0.4	up
	2,5-Dihydroxybenzaldehyde	18.1	266.9997	-0.3	down
	Tetradecane	19.3	57.0709	-0.7	down
	6,7-Dimethyl-1,2,3,5,8,8a-hexahydronaphthalene	20.5	91.0545	20.1	up
	α-Farnesene	27.6	57.0709	-0.2	down
6 d	p-Xylene	9.0	91.0054	19.9	up
	Eucalyptol	13.9	119.0848	1.5	up
	trans-β-Ocimene	14.2	145.9686	21.7	up
	β-Ocimene	14.6	145.9681	4.7	up
	2,4,6-Octatriene	17.2	121.1007	4.1	up
	Dodecane	20.6	57.0704	0.1	up
	n-Tridecan-1-ol	22.4	69.0700	-19.1	down
	1-Octanol	22.8	69.0700	0.8	up
	7-epi-Silphiperfol-5-ene	24.2	175.1478	1.8	up
	Caryophyllene	25.4	91.0538	0.2	up
	Butylated Hydroxytoluene	27.7	205.1583	0.5	up
9 d	trans-β-Ocimene	14.3	145.9686	2.4	up
	β-Ocimene	14.6	145.9681	1.9	up
	2,4,6-Octatriene	17.2	121.1007	2.1	up
	1,3-Cyclohexadiene	17.6	121.1022	18	up
	6,7-Dimethyl-1,2,3,5,8,8a-hexahydronaphthalene	20.6	91.0541	-0.7	down
	Nonadecane	22.2	57.0599	1.2	up
	1-Octanol	22.8	69.0700	0.2	up
	β-Guaiene	23.1	119.0856	0.1	up
	7-epi-Silphiperfol-5-ene	24.2	175.1478	1.0	up
	Silphiperfol-5-ene	24.3	175.1483	1.4	up
	tert-Hexadecanethiol	27.0	161.1327	2.0	up
	Butylated Hydroxytoluene	27.7	205.1583	0.8	up
	Naphthalene	28.0	159.1168	1.8	up
